# Post-Transcriptional Regulator Hfq Binds Catalase HPII: Crystal Structure of the Complex

**DOI:** 10.1371/journal.pone.0078216

**Published:** 2013-11-06

**Authors:** Koji Yonekura, Masahiro Watanabe, Yuko Kageyama, Kunio Hirata, Masaki Yamamoto, Saori Maki-Yonekura

**Affiliations:** 1 Biostructural Mechanism Laboratory, RIKEN SPring-8 Center, Harima Institute, Sayo, Hyogo, Japan; 2 Bio-Specimen Platform Group, RIKEN SPring-8 Center, Harima Institute, Sayo, Hyogo, Japan; 3 Research Infrastructure Group, RIKEN SPring-8 Center, Harima Institute, Sayo, Hyogo, Japan; Indian Institute of Science, India

## Abstract

We report a crystal structure of Hfq and catalase HPII from *Escherichia coli*. The post-transcriptional regulator Hfq plays a key role in the survival of bacteria under stress. A small non-coding RNA (sRNA) DsrA is required for translation of the stationary phase sigma factor RpoS, which is the central regulator of the general stress response. Hfq facilitates efficient translation of *rpoS* mRNA, which encodes RpoS. Hfq helps in the function of other specific proteins involved in RNA processing, indicating its versatility in the cell. However, structural information regarding its interactions with partners is missing. Here we obtained crystals of Hfq and HPII complexes from cell lysates following attempts to overexpress a foreign membrane protein. HPII is one of two catalases in *E. coli* and its mRNA is transcribed by an RNA polymerase holoenzyme containing RpoS, which in turn is under positive control of small non-coding RNAs and of the RNA chaperone Hfq. This sigma factor is known to have a pronounced effect on the expression of HPII. The crystal structure reveals that a Hfq hexamer binds each subunit of a HPII tetramer. Each subunit of the Hfq hexamer exhibits a unique binding mode with HPII. The hexamer of Hfq interacts via its distal surface. The proximal and distal surfaces are known to specifically bind different sRNAs, and binding of HPII could affect Hfq function. Hfq-HPII complexation has no effect on catalase HPII activity.

## Introduction

It is critically important for bacteria to survive environmental changes such as oxidative stress, heat/cold shock, iron excess, phosphosugar toxicity, and UV irradiation. The post-transcriptional regulator Hfq plays a key role in how bacteria cope with stress [1], [2] as well as in how hosts react to virulent pathogenic bacteria [3]. Hfq is a member of the family of Sm/Lsm proteins, which is widely distributed in both prokaryotes and eukaryotes. Eukaryotic Sm/Lsm proteins form heteroheptamers, and are involved in mRNA splicing and decay [4]–[6]. The bacterial Hfq family ranges in length from 70 to 110 amino acid residues and the proteins form thermostable homohexamers [2], [7], [8]. Hfq is abundant in *Escherichia coli*, with an estimated ∼10,000 copies of hexamers per cell [9]. The structures of all Hfq proteins are characterized by an N-terminal α-helix followed by a bent β-sheet composed of five anti-parallel strands and a flexible C-terminal segment [10]–[15]. The core structure without the C-terminal segment has the topology β5α1β1β2β3β4 and forms the hexamer primarily through intermolecular interactions between strands β4 and β5 of the neighbor. The hexameric toroid with an outer diameter of ∼70 Å and a thickness of ∼30 Å has two distinct faces [11], [12], [14], [15]. One side of the hexamer is named the proximal side and has a concave surface with radially projecting N-terminal α-helices, while the opposite distal side is convex and consists of β-sheets [11], [12], [14], [15].

Hfq helps small non-coding RNAs (sRNAs) base pair with their target mRNAs and regulate gene translation [2], [8], [16]. One of the best-studied examples is the post-transcriptional regulation of the stationary phase sigma factor σ^S^ (RpoS) [17], which is the central regulator of the general stress response in the stationary phase [18]. RpoS recognizes particular promoter sequences in DNA for gene transcription in this phase.

A sRNA, DsrA, consisting of 87 nucleotides, aids in translation initiation of rpoS mRNA [19], [20]. At low temperature the rpoS mRNA forms an intramolecular secondary structure that impedes translation initiation. DsrA anneals with rpoS mRNA sequences opposite of the ribosome binding site (RBS), which assists in maintaining the RBS in an open conformation and thus supports translation. Hfq facilitates this process by functioning as an RNA chaperone [17], [21].

The stationary phase sigma factor was initially identified as a factor that affects catalase activity in *E. coli*, and its gene was named *katF* [22]. Later, *katF* was found to encode a sigma factor, now recognized as RpoS, which regulates the transcription of the *katE* gene that encodes catalase HPII [23]. HPII is one of two catalases in *E. coli* and is expressed in the stationary phase [24]. RNA polymerase holoenzyme with RpoS transcribes *katE* mRNA as well as the mRNA of other stress-response proteins. This allows bacteria to survive under oxidative stress in the stationary phase.

Hfq is also involved in termination and destruction of mRNAs [2], [8], [16], [25]. Several studies have indicated that Hfq interacts with components of the ribosome, the RNA-decay machinery and/or other unknown proteins [2], [8], but structural information of interactions with other proteins is missing. Here, we report a crystal structure of Hfq and catalase HPII,both endogenous proteins of *E. coli*. The structure reveals that one Hfq hexamer binds each subunit of the HPII tetramer. Further, each subunit of the Hfq hexamer binds uniquely to HPII. Many of the residues on the distal face that interact with HPII are also known to interact with sRNA fragments.

## Results and Discussion

### Characterization of the crystal

**Figure 1:**
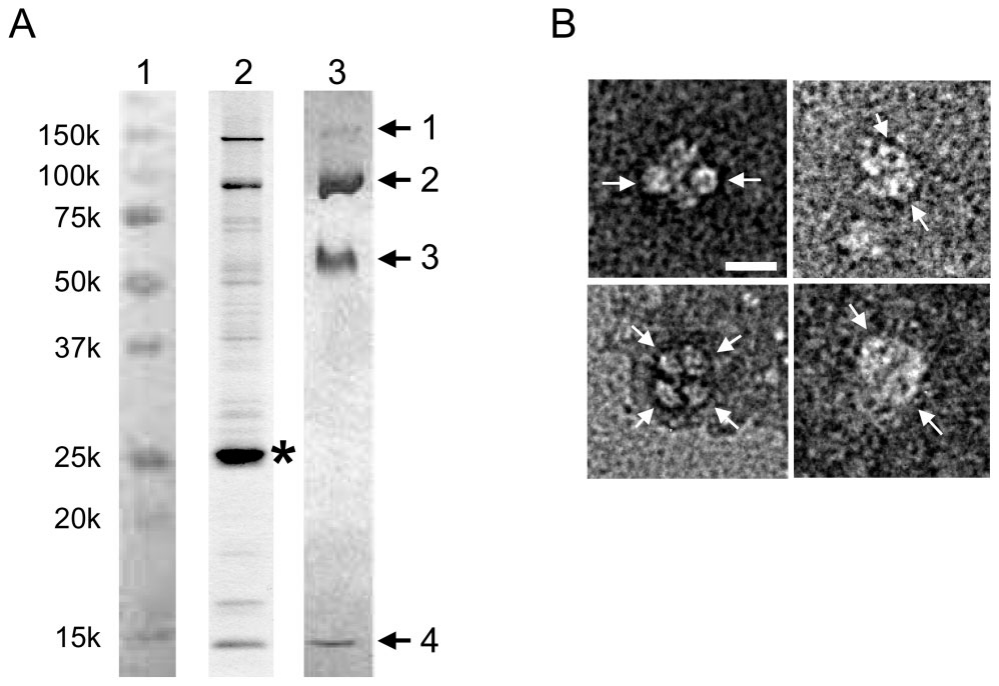
Characterization of protein samples. (A) An SDS-PAGE pattern of isolated protein solution and crystals of the Hfq and catalase HPII complex dissolved in SDS buffer solution. Lanes are: 1, markers; 2, protein solution after nickel-affinity chromatography; and 3, crystals of Hfq and HPII complex. Arrows and numbers in lane 3 indicate samples for PMF MALDI-TOF analysis. Asterisk in lane 2 indicates an overproduced PomA protein with a histidine tag. Bands 1 and 2 were identified as dimer and monomer of HPII and bands 3 and 4 as hexamer and monomer of Hfq by the PMF analysis, respectively. Note that the fraction eluting from the nickel-affinity column contained significant amounts of both Hfq and HPII. (B) Typical electron micrographs of complexes of endogenous Hfq and HPII prepared with negative staining. Some ring-like structures with dimensions of 70 – 80 Å are indicated with arrows. Bar indicates 100 Å.

Preparations containing Hfq and catalase HPII were obtained following attempts to express mutant proteins of a Vibrio flagellar motor protein PomAB [26], [27]. Cell lysates were processed and subjected to nickel-affinity and gel filtration to yield a concentrated protein solution of Hfq and catalase HPII. We obtained Hfq and HPII only from the constructs that perturbed cell growth following channel induction and overnight incubation. The induction probably increased stress, and likely promoted expression of catalase HPII from the *katE* gene through RpoS. Crystallization trials of the sample solution gave pale green crystals with dimensions of 40 – 100 µm (Fig. S1A in File S1); the color is typical of purified catalase HPII containing heme [28]. To identify the crystal contents, we performed peptide mass finger printing (PMF) MALDI-TOF analysis. Fig. 1A, lane 3 shows an SDS-PAGE gel of crystals dissolved in SDS buffer solution. From bands cut out of the gel, PMF analysis unambiguously identified two proteins, Hfq and catalase HPII, both of which are endogenous in *E. coli*. All the samples showed remarkably high identity scores for the two proteins and no other candidates were flagged in the SwissProt database by Mascot analysis [29]. Bands 1 and 2 were identified as catalase HPII. The molecular weight of HPII is 84 kDa, and bands 1 and 2 correspond to the dimer and monomer, respectively. The molecular weight of Hfq is 11.2 kDa and bands 3 and 4 are the hexamer and monomer of Hfq, respectively. The Hfq hexamer has a histidine cluster around the central pore, and these histidines presumably allowed complexes of Hfq and HPII to bind to the nickel-affinity resin, and be eluted with imidazole (lane 2 in Fig. 1A).

We assayed the activity of catalase within the crystals. The crystals decomposed hydrogen peroxide (H_2_O_2_) to H_2_O and O_2_ as shown by a decrease in absorbance at λ = 240 nm (Fig. S1B in File S1) [30]. Optical microscopic observation showed that the appearance of the crystals did not change much after the assay.

### Electron microscopy

We applied a few µl of the isolated protein solution onto a carbon-coated grid and negatively stained it. Electron microscopy (EM) showed large complexes with ring-like structures (Fig. 1B). The total dimension of the complexes is 150 – 160 Å. The diameter of the ring is 70 – 80 Å, which is consistent with that of the Hfq hexamer. Given the long-axis dimension of the rectangular HPII tetramer of ∼150 Å, the larger complexes with the rings are most likely Hfq hexamers bound to one HPII tetramer. We cannot tell the exact number of Hfq hexamers associated in each complex due to the limited resolution of the EM images. This observation also supports the view that complex formation takes place prior to crystallization.

### Binding of pure Hfq and pure catalase HPII *in vitro*

**Figure 2:**
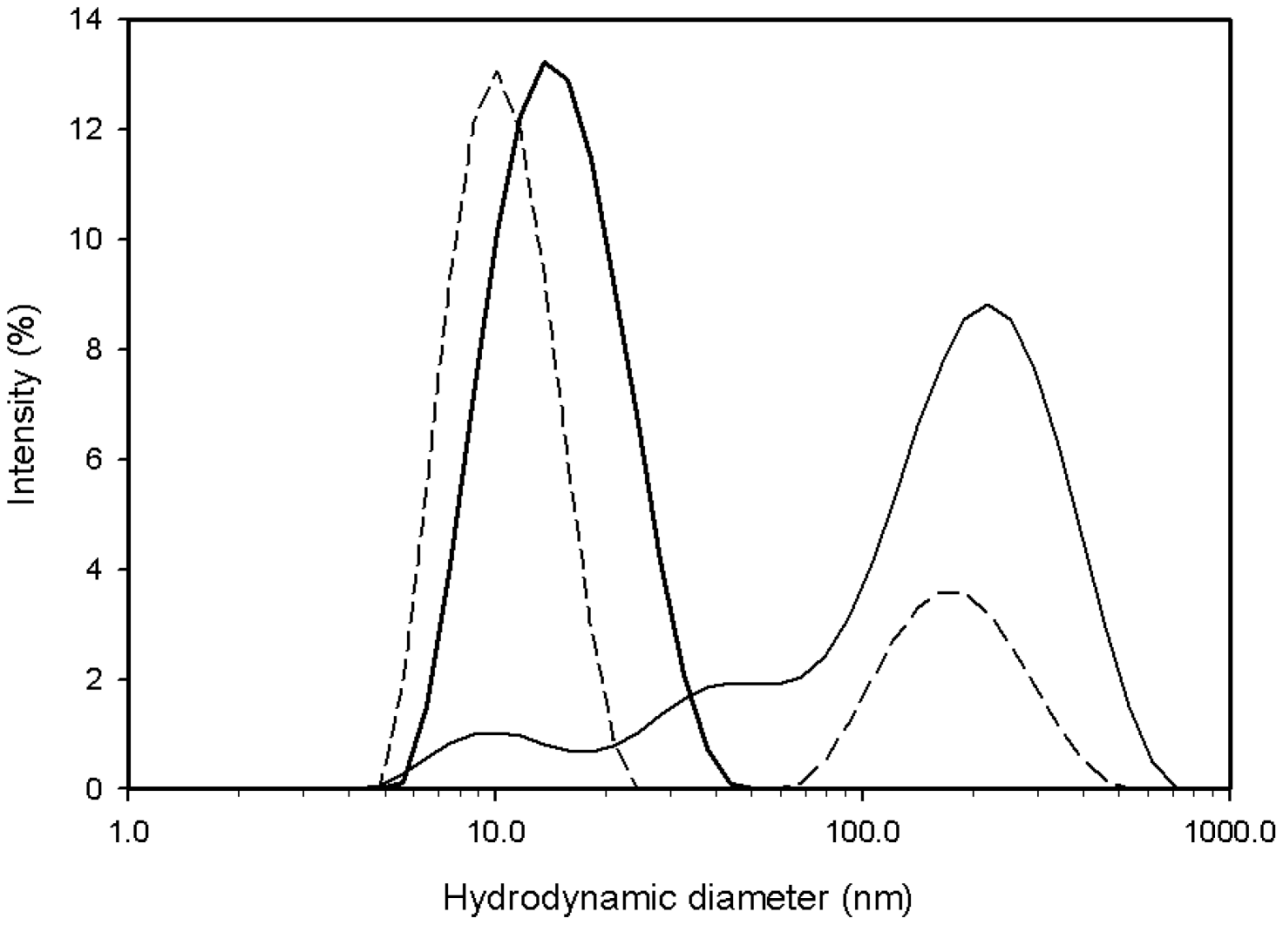
Intensity profiles of dynamic light scattering (DLS). Broken line: wild-type Hfq (∼1.0 mg/ml) after incubation at 40°C. Measured at 25°C. Thin line: mixture of wild-type Hfq (∼0.5 mg/ml) and HPII (∼0.5 mg/ml) kept at 4°C. Measured at 5°C. Thick line: the same sample as shown in thin line, but after incubation at 40°C. Measured at 25°C. The horizontal axis is common logarithm scale and the vertical axis is percent of the total intensity. The hydrodynamic diameter of the smaller peak in broken line is 9.8 nm corresponding to a molecular weight of 138 kDa, while that of the peak in thick line is 15.8 nm corresponding to ∼420 kDa.

To determine interactions of Hfq and HPII *in vitro*, we constructed over-expression systems for Hfq and HPII (SI Materials and methods in File S1) and purified them separately. Hfq needed to pass through an anion exchange column to remove bound nucleic acids [31]. Gel-filtration analysis (Fig. S2 in File S1) and dynamic light scattering (DLS; Fig. 2; see below) showed that purified HPII formed tetramers, whereas the Hfq hexameric rings formed dimers in buffer (150 mM NaCl and 20 mM Tris-HCl, pH8.0). Isothermal titration calorimetry (ITC) of injected concentrated Hfq into an HPII solution did not give clear signals of heat changes (SI Materials and methods in File S1). The dimer formation of the Hfq rings is probably through the distal side as in the crystal structure of full-length Hfq [15], the side which is the primary surface for complex formation with catalase HPII (see below). This could explain why ITC did not give clear signals of heat changes at room temperature. After mixing Hfq and HPII and incubating at 4°C overnight, gel filtration showed two separate peaks corresponding to the dimer of the Hfq ring and the HPII tetramer (Fig. S2 in File S1). However, after incubation of Hfq and HPII at 40°C for an hour, the peak fraction of size-exclusion chromatography contained both Hfq and HPII (Fig. S2 in File S1). EM of this fraction resolved complexes with ring-like structures (Fig. S3 in File S1).

DLS showed that the sample solution of Hfq contained one primary species and aggregations or large oligomers (broken line in Fig. 2). The hydrodynamic diameter of this main species is 9.8 nm, which corresponds to a molecular weight of 138 kDa. It is consistent with the dimer of the Hfq hexamer. When Hfq and HPII were mixed and kept at 4°C, DLS gave patterns indicative of aggregates / large oligomers (thin line in Fig. 2). Incubation at 40°C reduced the amount of aggregates and produced complexes with a hydrodynamic diameter of 15.8 nm, which corresponds to a molecular weight of ∼420 kDa (thick line in Fig. 2). The peak was broad, suggesting multiple species of complexes (thick line in Fig. 2). Considering the molecular weights of Hfq and HPII, these complexes were probably composed of 1 ∼2 Hfq hexamers and one HPII tetramer.

The higher temperature may facilitate dissociation of hexamer dimers, thereby promoting exchange of a Hfq hexameric ring for HPII. However a high temperature does not seem necessary for complex formation in the cell, since we apparently obtained ample formation in our cells grown at 30°C. Hfq-HPII complexes were found in cells stressed by the induction of a foreign membrane protein in an introduced plasmid, which presumably led to the overexpression of HPII. Thus stress and overproduction of HPII may be the physiological cue for complex formation in cells.

### Overall structure of the Hfq and HPII complex

**Figure 3:**
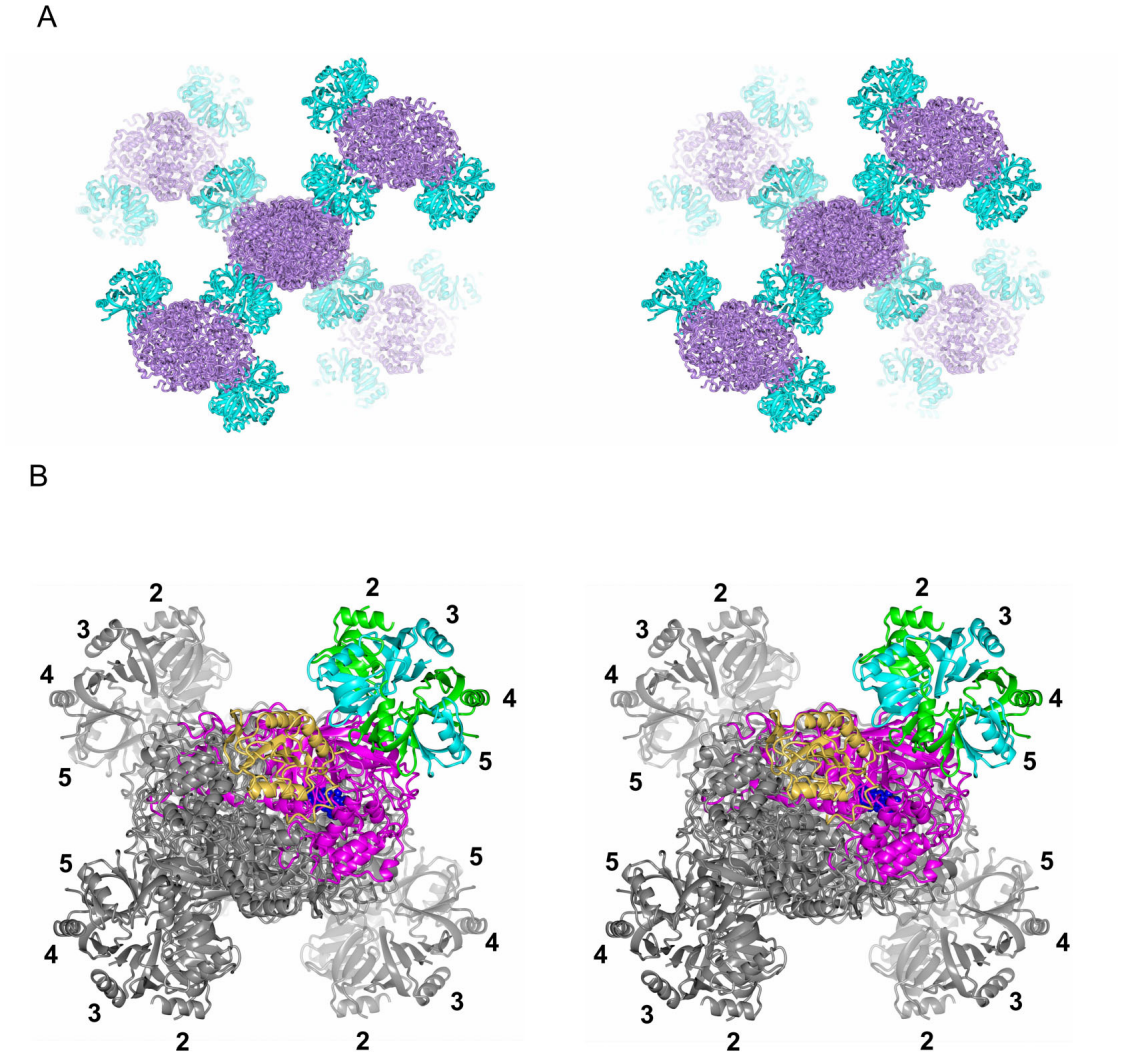
Crystal structure of the Hfq and catalase HPII complex. (A) Stereo diagram showing the crystal packing of the complex composed of Hfq hexamers in cyan and HPII tetramers in violet. All the four bound Hfq hexamers are displayed for the HPII tetramer at the center, whereas only two hexamers are displayed for each surrounding HPII tetramer for clarity. (B) Structure of one HPII tetramer with four bound Hfq hexamers showing interaction through their distal surfaces. Viewed in stereo. Subunits of one Hfq hexamer are displayed in cyan and green. Numbers 2 – 5 indicate the subunit number in the Hfq hexamer as in Fig. 4. One molecule of the HPII tetramer is displayed in tan (the C-terminal lobe) and in magenta (the other parts). A space-filling model in blue represents heme. Other models are in grey.

**Figure 4:**
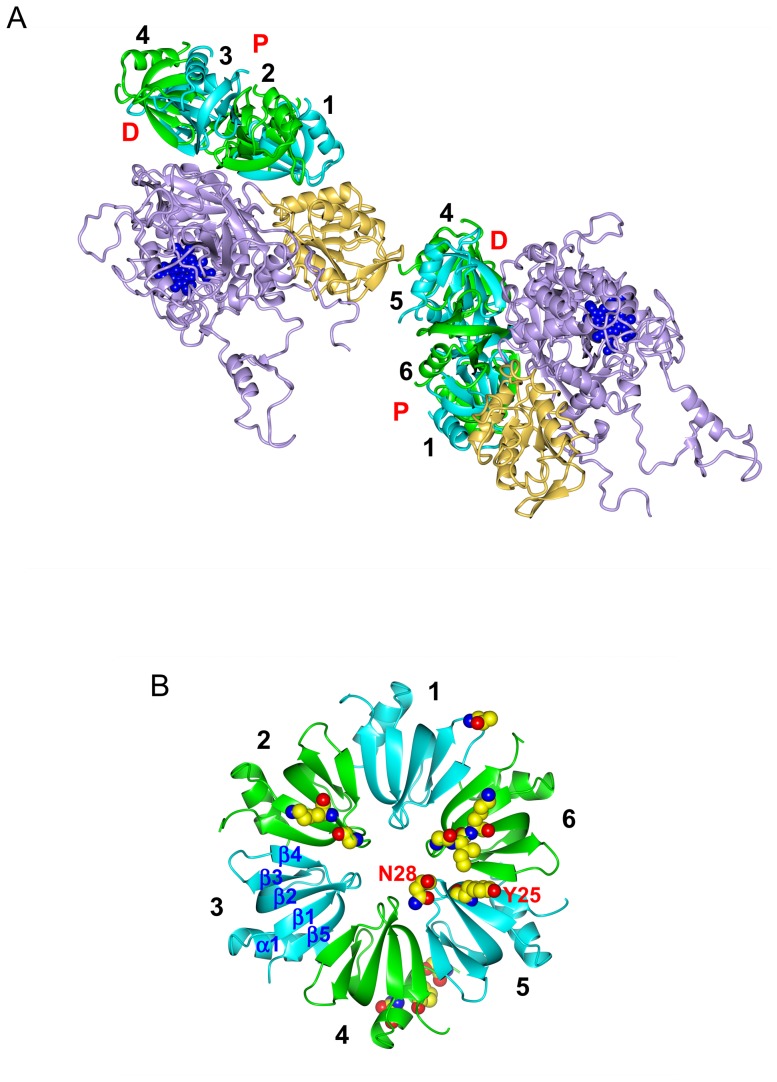
Some structural details. (A) Two HPII molecules and their interaction partners, two Hfq hexamers. Hfq subunits are color-coded as in Figs. 3B and 4B, and HPII is displayed in tan (the C-terminal lobe) and in violet (the other parts) with heme in blue. “P” and “D” denote the proximal and distal sides of the Hfq hexamer, respectively. (B) Hfq hexamer viewed from the distal side. Residues for binding to HPII are drawn in space-filling representation (see also Table S2 in File S1) with the single-letter amino acid code for Tyr 25 and Asn 28 in subunit 5 (see Fig. 5). Atoms are color-coded as: carbon, yellow; nitrogen, blue; and oxygen, red. “α1” denotes the N-terminal α-helix and “β1” – “β5” β-strands. Numbers 1 - 6 in A and B indicate the subunit number in the Hfq hexamer. Only the Hfq subunits on the front side have the number in A for clarity.

Crystals were mounted on a microfocus beam line (BL32XU) at SPring-8 and showed good diffraction of X-rays [32]. We solved the crystal structure at 2.85-Å resolution by molecular replacement with R_work_ and R_free_ values of 19.9% and 24.1%, respectively. Data statistics are shown in Table S1 in File S1. Fig. 3 shows the crystal structure of the complex. Catalase HPII is tetrameric, as in the crystals of pure catalase [33], [34], and one Hfq hexameric ring is bound to each corner of the HPII tetramer, one per each individual HPII subunit. The Hfq ring sits on the central core domain of HPII and leans onto a lobe at the C-terminus (Fig. 4A). The core domain of *E. coli* HPII consists of amino acids roughly from 120 to 520 and this domain is conserved from prokaryotes to mammals, whereas the C-terminal lobe consisting of amino acids from roughly 595 to the C-terminal end is variable between species, and bovine catalase has no such C-terminal domain [33].

Approximately 40 residues at the C-terminus of Hfq were missing in the electron density map. This is a common feature in the crystal structure of pure Hfq [15], and consequently the C-terminal region is considered flexible and disordered [11], [15]. Formation of the complex does not change the conformations of the HPII tetramer nor the Hfq hexamer. The root-mean-square deviation (RMSD) of Cαs in the Hfq hexamer is ∼0.59 Å (total 377 residues in the hexamer) and that in the HPII tetramer is ∼0.28 Å from the starting models [15], [34].

The packing in the crystal is shown in Fig. 3A. There are no direct contacts between different HPII tetramers nor between different Hfq hexamers. All the contacts are restricted to the interface between Hfq and HPII. To clarify the interactions, we cut out two HPII molecules from adjacent tetramers and two Hfq hexamers as in Fig. 4A. Most of interactions are formed between the distal surface of the Hfq hexamer and the conserved core domain of HPII. We calculated that the buried solvent-accessible surface area (SASA) between this side of one Hfq ring and one HPII molecule is 1066.7 Å^2^. This value is not large, but would be significant for the complex formation (cf. the buried SASA between a diagonal pair of two HPII molecules in the HPII tetramer is 2014.7 Å^2^). The Hfq ring structure fits well onto the concave surface of HPII (Fig. 4A). H_2_O_2_ enters HPII from the lateral side of the molecule, binding to heme at the center of HPII [33]. The Hfq hexamer attached on HPII does not sterically hinder this path (Figs. 3 and 4A) [33]. Thus, the association of the Hfq hexamer with HPII does not interfere with the measured catalase activity (Fig. S1B in File S1).

In contrast, clear contacts on the proximal side of Hfq are limited. We only found a few interactions between N-terminal residues in one Hfq subunit and the lower part of the C-terminal lobe of a neighboring HPII tetramer (Fig. 4A), and categorized these interactions as crystal contacts. This part of the C-terminal lobe is opposite to the surface that interacts with the distal surface of Hfq (Fig. 4A). The flexible C-terminal domain of Hfq may contribute to crystal contacts as presumed from the crystal packing of full-length Hfq [15].

### Interactions between Hfq and HPII

**Figure 5:**
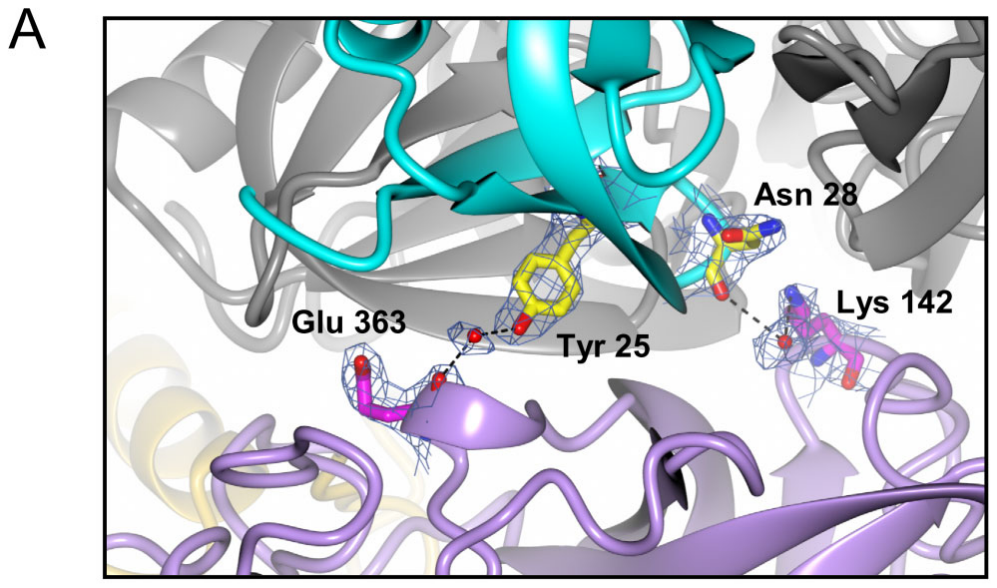
Interactions between Hfq subunit 5 and HPII. Subunit 5 is displayed in cyan and one molecule of the HPII tetramer is color-coded as in Fig. 4A. Other models are displayed in grey. Interacting residues are drawn in ball-and-stick representation overlaid with 2 Fo - Fc maps of 1.0 σ, and atoms are color-coded as: nitrogen, blue; oxygen, red; and carbon, yellow in Hfq and magenta in HPII. Bonds are depicted as black dashes. Interactions of subunits 2 and 6 with HPII are shown in Fig. S4 in File S1 and those of subunits 1 and 4 are in Fig. S5 in File S1. See also Table S2 in File S1 for bond length and type.

Interactions between each Hfq subunit and HPII are unique. To describe the details of the interactions we numbered each Hfq subunit 1 – 6 as in Fig 4B. Subunit 1 is defined as the closest to the C-terminal lobe of HPII. When viewed from the distal side, subunits 2 – 6 were assigned anticlockwise (Fig. 4B). Each subunit has contacts with HPII except for subunit 3, which does not interact with HPII. Between the distal surface of Hfq and HPII, we found that residues for RNA binding [12], [14] make interactions with HPII. Those are Gly 29 in Hfq subunit 2 - Asn 157 in the HPII core domain; Gly 29 (subunit 6) - Arg 369 (the HPII core domain); Lys 31 (subunit 2) - Asn 157 (the core domain); Lys 31 (subunit 6) - Pro 295 (the core domain); Tyr 25 (subunit 5) - Glu 363 (the core domain); Asn 28 (subunit 5) - Lys 142 (the core domain); and Ile 30 (subunit 6) - Pro 366 (the core domain). Figs. 5 and S4 in File S1 show close-up views of contacts between the distal surface of subunits 2, 5, 6 and the core domain of HPII. The interface between subunit 1 and the C-terminal lobe of HPII is shown in Fig. S5A in File S1.

The distal surface of the Hfq ring binds adenosine (A)-rich RNAs [12]. There is an adenosine-binding pocket called the A site and another pocket for a purine nucleotide, guanosine or adenosine, called the R site [12]. Lys 31, Leu 32, Gln 33 and Gln 52 from β-strands 2 and 4 primarily form the A site [12], while Tyr 25, Asn 28, Gly 29 and Ile 30 are involved in binding of an adenosine in short RNA fragments at the R site [12], [14].

On the proximal side, N-terminal residues in subunit 4 exclusively contribute to the interaction with a HPII molecule in a different tetramer (Fig. 4A). Those include Ser 6 - Gly 645 in the C-terminal lobe of a neighboring HPII molecule; Asn 13 - Glu 610 (the C-terminal lobe); and Pro 10 - Asp 644 (the C-terminal lobe). Most of the proximal side of the hexamer faces the solvent (Figs. 3A and 4A). Fig. S5B in File S1 shows a close-up view of the interface between the proximal side of subunit 4 and the lower C-terminal lobe of HPII. Table S2 in File S1 has more information on major interactions.

Crystal structures of Hfq with bound RNA reveal that residues around the central pore on the proximal side are responsible for binding of U-rich sRNA segments [10], [13], [14]. Ser 6, Gln 8 and Asp 9 are involved in a novel uridine-recognition site [14]. Thus complexes of Hfq and HPII could still bind U-rich sRNAs through the exposed proximal side of the Hfq hexamer, as long as the disordered C-terminal domain of Hfq does not occupy this space. The role of the C-terminal domain of Hfq is not clear, but a recent study suggested that this domain also interacts with RNA, which could increase binding specificity [35].

Given that the proximal and distal surfaces of the Hfq ring bind different types of sRNA sequences, complex formation with HPII would limit its repertoire for binding sRNAs in the cell, and regulate its chaperone function. Further studies are needed to explore this exciting possibility.

## Materials and Methods

### Samples preparation and crystallization

Complexes of Hfq and catalase HPII were obtained fortuitiously when overexpressing mutants of a bacterial sodium channel protein called PomAB [26], [27]. Plasmids containing the corresponding mutant genes fused with a hexahistidine tag sequence were expressed under the control of the T7 promoter. We found that mutants causing a slight disruption to cell growth after induction by IPTG produced a significant amount of the Hfq and catalase HPII complex following overnight incubation at 30°C and isolation by nickel-affinity chromatography. Complexes of Hfq and HPII were found in samples obtained from the cell lysate by nickel-affinity and gel-filtration chromatography and were suspended in 10 mM Tris-HCl (pH 8.0), 50 mM NaCl, 10% (v/v) glycerol, 1 mM DTT and 0.2% (w/v) Cymal5 (Anatrace). Crystals were grown by hanging-drop vapor diffusion at 20°C in a mother liquor containing 0.1 M Tris-HCl (pH 9.0), 0.18 M NaCl and 10% PEG 4000. Pale green crystals grew to 40 – 100 µm over a month (Fig. S1A in File S1). More details are described in SI Materials and methods in File S1.

### Protein identification with PMF MALDI-TOF

We collected crystals with a nylon loop and washed them with buffer solution. The crystals were dissolved in SDS sample buffer, heated at ∼95°C for 5 min, and then run on a 12.5% homogeneous polyacrylamide gel. Bands cut out from the gel stained with Coomassie brilliant blue were digested with trypsin. The digestion mixtures were subjected to MALDI-TOF MS on an Ultraflex mass spectrometer (Bruker Daltonics) followed by PMF analysis. The obtained MS spectra were used to search the SwissProt 57.15 database using the Mascot program [29], which identified two proteins, Hfq and catalase HPII, and no others.

### Electron microscopy

A few *µ*l of the peak fraction of gel filtration was applied onto a carbon-coated copper grid and negatively stained with 2% uranyl acetate. We examined the sample grids with a JEOL-2100 electron microscope (JEOL) equipped with a LaB_6_ gun operated at an accelerating voltage of 200 kV. Images were recorded on a slow-scan charge-coupled device (SSCCD) camera (MegaScan, Gatan) at a final magnification of ∼65,000. The magnification was calibrated with negatively stained bovine catalase crystals.

### Catalase assay

We selected 5 ∼6 crystals from a crystallization plate, washed them with buffer solution, and transferred them into 50 µL of the buffer solution in a 384-well flat-bottom UV-transparent microtiter plate. A 0.5 µL volume of 3% hydrogen peroxide was added to the well of the microtiter plate, and the plate was then immediately scanned in a Varioskan Flash spectrophotometer (Thermo Scientific) [30] at λ = 240 nm every 1 s for 5 min at room temperature. Catalase decomposes hydrogen peroxide, which has an absorbance peak at λ = 240 nm, and we measured the decrease in absorbance for an indication of catalase activity.

### *In vitro* binding assay of Hfq and HPII

We cloned the catalase *HPII* and Hfq genes from *E. coli* chromosome, and constructed *Hfq* mutants (SI Materials and methods in File S1). The target proteins were purified by nickel-affinity and anion-exchange chromatography (SI Materials and methods in File S1). Isothermal titration calorimetry (ITC) and dynamic light scattering (DLS) of purified Hfq and HPII were done as in SI Materials and methods in File S1. We carried out analytical gel filtration of Hfq, HPII and mixtures of Hfq and HPII on a Superdex 200 5/150 GL column (GE Healthcare).

### Data collection and structure refinement

Crystals ware soaked quickly in glycerol solution by increasing the glycerol concentration stepwise up to 30% and flash-frozen in cold nitrogen gas. Diffraction data were collected on a micro-focus beam line BL32XU of SPring-8 at a wavelength of 1 Å [32]. X-ray diffraction from whole crystals showed high diffuse backgrounds and blurred diffraction spots. A focused beam with a dimension of 1.6×10 µm gave excellent patterns. A total of 180° of diffraction frames were collected in 1° oscillations by shifting the sample by 1 µm every 3 frames. The data were processed to 2.85-Å resolution with HKL2000 [36]. The structure was solved by molecular replacement using PHASER [37], starting from an atomic model of an Hfq hexamer (PDB accession code: 3QHS; 15) and a model of a catalase monomer cut out from an atomic-coordinate file (accession code: 1GGE; 34). The crystals belonged to space group I222 (unit cell dimensions of *a* = 136.4 Å, *b* = 159.0 Å and *c* = 167.2 Å) and contained one Hfq hexamer and one HPII monomer in the asymmetric unit. The structure of Hfq - HPII was refined using REFMAC [38], and manually adjusted with COOT [39]. The stereochemical properties of the models were checked with PROCHECK [40] and the validation tools of COOT. The electron density map resolved amino acids 27 – 753 and one heme molecule for catalase HPII, and 6 – 68 (subunit 1; Fig. 4B), 7 – 69 (subunit 2), 5 – 66 (subunit 3), 5 – 67 (subunit 4), 5 – 67 (subunit 5) and 6 – 68 residues (subunit 6) for the Hfq hexamer. The map did not show ∼40 residues at the C-terminus of Hfq. In addition, we were able to pick up 29 oxygen atoms of water in the asymmetric unit. All the figures of molecular graphics were created with CCP4MG [41]. Data collection and refinement statistics are shown in Table S1 in File S1. The atomic coordinates and X-ray diffraction data have been deposited in PDB (code 3VU3).

## Supporting Information

File S1 Fig. S1, Crystal of the Hfq and catalase HPII complex and catalase activity of crystals. (A) A typical crystal for X-ray diffraction experiments. Bar represents 100 µm. (B) Assay curve for crystals of the Hfq and catalase HPII complex. Decomposition of hydrogen peroxide (H_2_O_2_) over time shows the decrease in absorbance at λ = 240 nm. Time = 0 indicates a point just after H_2_O_2_ was added. Fig. S2, Analytical gel-filtration of Hfq and HPII. (A) Dashed line: wild-type Hfq after incubation at 40°C. Thin line: mixture of wild-type Hfq (∼0.5 mg/ml) and HPII (∼0.5 mg/ml) at 4°C. Thick line: the same sample as shown in the thin line, but after incubation at 40°C. The peak in the dashed line corresponds to 138 kDa and the earlier eluting peak at ∼1.7 ml in thin line corresponds to ∼320 kDa as calibrated with soluble globular proteins used as standards. (B) SDS-PAGE patterns of overproduced and purified Hfq and HPII. Lanes are: 1, marker; 2, Hfq; 3, HPII; and 4, the peak fraction of analytical gel-filtration of mixture of Hfq and HPII shown in the thick line in (A). Arrows are: upper, HPII; middle, hexamer of Hfq; and lower, monomer of Hfq. Other bands probably indicate degradation of HPII after incubation at 40°C. Fig. S3, Typical electron micrographs of protein complexes prepared with negative staining of the peak fraction in thick line in Fig. S2. Purified Hfq and HPII were mixed and incubated at 40°C and gel-filtrated. Protein complexes with ring-like structures are similar to complexes of endogenous Hfq and HPII (Fig. 1B). Bar refers to 100 Å. Fig. S4, Interactions of Hfq subunits 2 and 6 with HPII. (A) Subunit 2 in green. (B) Subunit 6 in green. The figures display the interface between the distal surface of Hfq and the core domain of HPII. The representation scheme of molecules is the same as in Fig. 5. See also Table S2 for more information on the interactions. Fig. S5, Interactions of Hfq subunits 1 and 4 with HPII. (A) Interface between the distal surface of Hfq subunit 1 (cyan) and the C-terminal lobe of HPII. (B) Interface between the proximal surface of Hfq subunit 4 (green) and lower part of the C-terminal lobe of HPII in a neighboring tetramer. The representation scheme is the same as in Fig. 5. Densities for the side chains of Glu 610 are not visible at this contour level. Table S1, Data collection and refinement statistics for the *E.coli* Hfq-catalase HPII complex. Table S2, Major interactions between Hfq and catalase HPII. SI Materials and methods.
(PDF)
