# Supplementary material for: Post-Transcriptional Regulator Hfq Binds Catalase HPII: Crystal Structure of the Complex
Source: PLoS One. 2013 Nov 6;8(11):e78216. doi: 10.1371/journal.pone.0078216 (PMC3819363; doi:10.1371/journal.pone.0078216)
Supplement: File S1 — Fig. S1, Crystal of the Hfq and catalase HPII complex and catalase activity of crystals. (A) A typical crystal for X-ray diffraction experiments. Bar represents 100 µm. (B) Assay curve for crystals of the Hfq and catalase HPII complex. Decomposition of hydrogen peroxide (H2O2) over time shows the decrease in absorbance at λ = 240 nm. Time = 0 indicates a point just after H2O2 was added. Fig. S2, Analytical gel-filtration of Hfq and HPII. (A) Dashed line: wild-type Hfq after incubation at 40°C. Thin line: mixture of wild-type Hfq (∼0.5 mg/ml) and HPII (∼0.5 mg/ml) at 4°C. Thick line: the same sample as shown in the thin line, but after incubation at 40°C. The peak in the dashed line corresponds to 138 kDa and the earlier eluting peak at ∼1.7 ml in thin line corresponds to ∼320 kDa as calibrated with soluble globular proteins used as standards. (B) SDS-PAGE patterns of overproduced and purified Hfq and HPII. Lanes are: 1, marker; 2, Hfq; 3, HPII; and 4, the peak fraction of analytical gel-filtration of mixture of Hfq and HPII shown in the thick line in (A). Arrows are: upper, HPII; middle, hexamer of Hfq; and lower, monomer of Hfq. Other bands probably indicate degradation of HPII after incubation at 40°C. Fig. S3, Typical electron micrographs of protein complexes prepared with negative staining of the peak fraction in thick line in Fig. S2. Purified Hfq and HPII were mixed and incubated at 40°C and gel-filtrated. Protein complexes with ring-like structures are similar to complexes of endogenous Hfq and HPII (Fig. 1B). Bar refers to 100 Å. Fig. S4, Interactions of Hfq subunits 2 and 6 with HPII. (A) Subunit 2 in green. (B) Subunit 6 in green. The figures display the interface between the distal surface of Hfq and the core domain of HPII. The representation scheme of molecules is the same as in Fig. 5. See also Table S2 for more information on the interactions. Fig. S5, Interactions of Hfq subunits 1 and 4 with HPII. (A) Interface between the distal surface of Hfq subu [file pone.0078216.s001.pdf]

Supporting Information

Post-transcriptional regulator Hfq binds catalase HPII: crystal structure of the complex

Koji Yonekura, Masahiro Watanabe, Yuko Kageyama, Kunio Hirata, Masaki Yamamoto and Saori Maki-Yonekura

SI Materials and methods

Sample preparation and crystallization of the Hfq and catalase HPII complex

Preparations of Hfq and catalase HPII complexes were obtained following attempts to express mutant proteins of a Vibrio flagellar motor protein PomAB. The plasmids carrying PomAB mutants fused with a hexahistidine (His6) tag sequence were transformed in *E. coli* BL21 (DE3) Gold competent cells (Novagen) and expressed under the control of the T7 promoter. The cells were grown in Luria–Bertani (LB) medium with 50 μg ml/l ampicillin to an absorbance at 600 nm of 0.6 ~ 0.8. After adding IPTG to a concentration of 0.2 mM, the cells were incubated overnight at 30 °C and harvested by centrifugation. The pellet was lysed with ~ 7 passages at ~ 10,000 psi in an EmulsiFlex-C5 high-pressure homogenizer (Avestin). The lysate was subjected to centrifugation at 200,000 g for 1.5 h and the pellet resuspended in 20 mM Tris-HCl (pH 8.0), 150 mM NaCl, 10% (v/v) glycerol, 1% (w/v) Cymal-5 (Anatrace) and 1 tablet of 'Complete EDTA-free' protease inhibitor cocktail (Roche Applied Science). The suspension was held at room temperature for 1 h, after which it was centrifuged at 100,000 g for 1 h. The supernatant was applied to a column filled with Ni-NTA agarose (QIAGEN) and the column was allowed to stand at 4°C for 1 h. The column was washed with the suspension buffer except, in addition, containing 0.2% Cymal-5

(Anatrace) and 10 mM imidazole. The column was developed with a gradient of imidazole and fractions eluting at ~ 300 mM imidazole were collected. The eluate was concentrated with a spin concentrator with a molecular weight cut-off of 50,000 and applied to a gel filtration column, Superdex 200 10/300 GL (GE Healthcare). It was run at a flow rate of 0.3 - 0.4 ml/min in the same buffer but not containing imidazole. A fraction at the main peak was collected, passed through an Econo-Pac 10DG desalting column (BioRad) with 10 mM Tris-HCl (pH 8.0), 50 mM NaCl, 10% (v/v) glycerol, 0.2% Cymal-5 (Anatrace) and 1 mM DTT, and concentrated to a total protein concentration of 3 ~ 7 mg/ml. Crystals of the Hfq and catalase HPII complex were grown from the sample solution by hanging-drop vapor diffusion at 20°C in a mother liquor containing 0.1 M Tris-HCl (pH 9.0), 0.18 M NaCl and 10% PEG 4000.

Cloning of the catalase *HPII* gene

The *E.coli* catalase *HPII* gene (*katE*) was amplified from genomic DNA of *E.coli* K12 strain by polymerase chain reaction (PCR) using the forward primer 5’GGGCCCCATATGTCGCAACATAACGAAAAGAACCC3’ and the reverse primer 3'GGGCCCCTCGAGTCAGGCAGGAATTTTGTCAATC5’. The PCR product was purified by illustra bacteria genomicPrep mini spin kit (GE Healthcare). The gene was cloned into pET28b vector between the Nde I and Xho I sites with the His6 tag at the N terminus and a stop codon (TGA). The product was verified by sequencing.

Cloning of the *hfq* gene and construction of *hfq* mutants

The *E.coli hfq* gene was amplified from genomic DNA of *E.coli* K12 strain by PCR using the forward primer 5’GGGCCCCATATGTCGCAACATAACGAAAAGAACCC3’

and the reverse primer 3’GGGCCCCTCGAGTCAGGCAGGAATTTTGTCAATC5’. The PCR product was purified by illustra bacteria genomicPrep mini spin kit (GE Healthcare). The gene was cloned into pET28b vector between Nde I and Xho I sites with the His6 tag at N terminus and a stop codon (TAA). Mutagenesis of Y25A, I30E, K31A, T49E was carried out using QuikChange Lightning site-directed mutagenesis kit (Agilent Technologies). The primers for the point mutations were:
5’CGTGTTCCAGTTTCTATTGCATTGGTGAATGGTATTAAGCTGC3’ (Y25A),
5’CTATTTATTTGGTGAATGGTGAGAAGCTGCAAGGGCAAATCG3’ (I30E),
5’CTATTTATTTGGTGAATGGTATTGCACTGCAAGGGCAAATCG3’ (K31A),
5’GTTCGTGATCCTGTTGAAAAACGAGGTCAGCCAGATGGTTTAC3’ (T49E). The products were verified by sequencing.

Isothermal titration calorimetry and dynamic light scattering

Catalase HPII and Hfq were dialysed against 20 mM Tris HCl pH 8.0 and 150 mM NaCl, and concentrated to 1.0 mg/ml (12 μM catalase monomer) and 10 mg/ml (160 μM Hfq hexamer ring), respectively. Then, the samples were passed through 0.22 μm filters and placed on a tray of a MicroCal Auto-iTC200 calorimeter (GE Healthcare). Heat changes were measured by injecting the Hfq solution from a syringe into the HPII solution in the sample cell.

Dynamic light scattering of Hfq, HPII and mixtures of Hfq and HPII was measured with a Zetasizer Nano S instrument (Malvern).

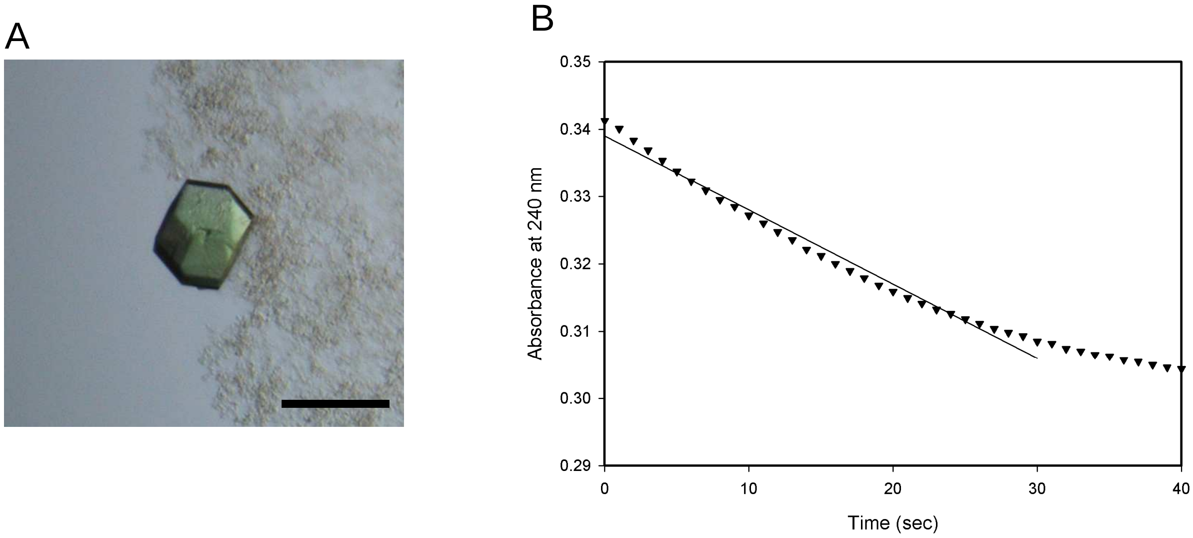

Fig. S1. Crystal of the Hfq and catalase HPII complex and catalase activity of crystals. (A) A typical crystal for X-ray diffraction experiments. Bar represents 100 μm. (B) Assay curve for crystals of the Hfq and catalase HPII complex. Decomposition of hydrogen peroxide (H_2O_2) over time shows the decrease in absorbance at λ = 240 nm. Time = 0 indicates a point just after H_2O_2 was added.

Table S1. Data collection and refinement statistics for the *E.coli* Hfq-catalase HPII complex

Space group	*I* 222
Unit cell	a = 136.4 Å b = 159.0 Å c = 167.2 Å
Resolution	50.0 – 2.85 Å
No. of reflections	189,054
No. of unique reflections	40,349
R_{merge} (%)	11.0 (30.1) #
Completeness (%)	95.5 (89.9) #
I/σ	8.6 (2.1) #
Redundancy	4.8 (3.1) #
Refinement	
Resolution range	20.0 – 2.85
R factor (%)	19.6 (27.6) †
Free *R* factor (%)	24.6 (32.0) †
Average B factor (Overall)	47.5
Rmsd bond lengths	0.013
Rmsd bond angles	1.9
Ramachandran plot	
Most favoured region (%)	94.8
Additionally allowed region (%)	4.3
Disallowed region (%)	0.9

# Numbers in brackets refer to the outer shell 2.90 – 2.85 Å.
† Numbers in brackets refer to the outer shell 2.93 – 2.85 Å.

Table S2. Major interactions between Hfq and catalase HPII

Hfq	HPII	Bond length (Å)	Bond type
Subunit 1			
Thr 49 CG2	Ala 737 CB	3.3	van der Waals
Thr 49 O	Lys 626 NZ	3.6 / 3.8	Water-mediated
Subunit 2			
Gly 29 O	Asn 157 ND2	3.4	hydrogen bond
Lys 31 N	Asn 157 OD1	3.7	hydrogen bond
Subunit 4	Neighbor #		
Ser 6 N	Gly 645 O	3.1	hydrogen bond
Pro 10 CG	Asp 644 C	3.4	van der Waals
Asn 13 ND2	Glu 610 OE2	3.4	hydrogen bond
Subunit 5			
Tyr 25 OH	Glu 363 O	3.5 / 3.7	Water-mediated
Asn 28 O	Lys 142 NZ	3.9 / 3.4	Water-mediated
Subunit 6			
Gly 29 O	Arg 369 N	2.6	hydrogen bond
Ile 30 CD1	Pro 366 CG	3.4	van der Waals
Ile 30 CG2	Pro 366 CB	3.6	van der Waals
Lys 31 NZ	Pro 295 O	3.9 / 3.5	Water-mediated

HPII molecule in the neighbor tetramer.

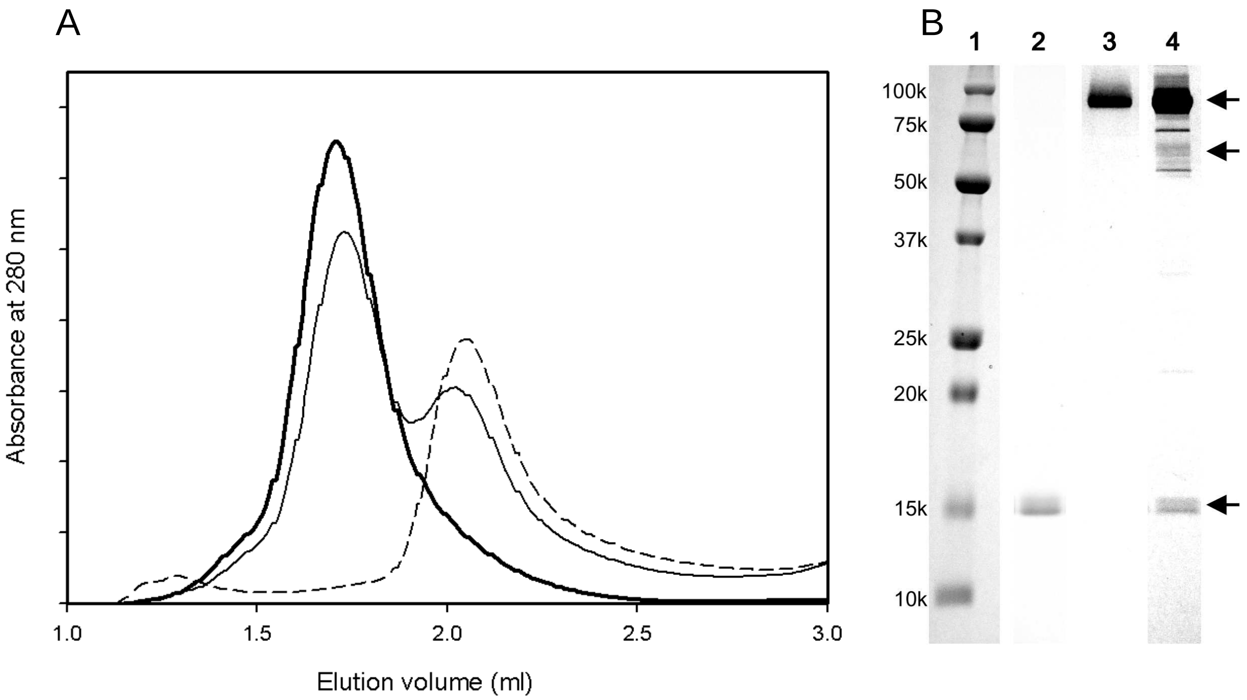

Fig. S2. Analytical gel-filtration of Hfq and HPII. (A) Dashed line: wild-type Hfq after incubation at 40°C. Thin line: mixture of wild-type Hfq (~ 0.5 mg/ml) and HPII (~ 0.5 mg/ml) at 4°C. Thick line: the same sample as shown in the thin line, but after incubation at 40°C. The peak in the dashed line corresponds to 138 kDa and the earlier eluting peak at ~ 1.7 ml in thin line corresponds to ~ 320 kDa as calibrated with soluble globular proteins used as standards. (B) SDS-PAGE patterns of overproduced and purified Hfq and HPII. Lanes are: 1, marker; 2, Hfq; 3, HPII; and 4, the peak fraction of analytical gel-filtration of mixture of Hfq and HPII shown in the thick line in (A). Arrows are: upper, HPII; middle, hexamer of Hfq; and lower, monomer of Hfq. Other bands probably indicate degradation of HPII after incubation at 40°C.

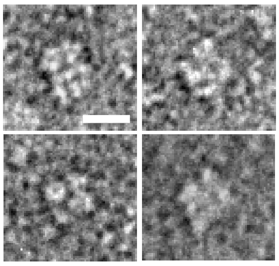

Fig. S3. Typical electron micrographs of protein complexes prepared with negative staining of the peak fraction in thick line in Fig. S2. Purified Hfq and HPII were mixed and incubated at 40°C and gel-filtrated. Protein complexes with ring-like structures are similar to complexes of endogenous Hfq and HPII (Fig. 1B). Bar refers to 100 Å.

A

B

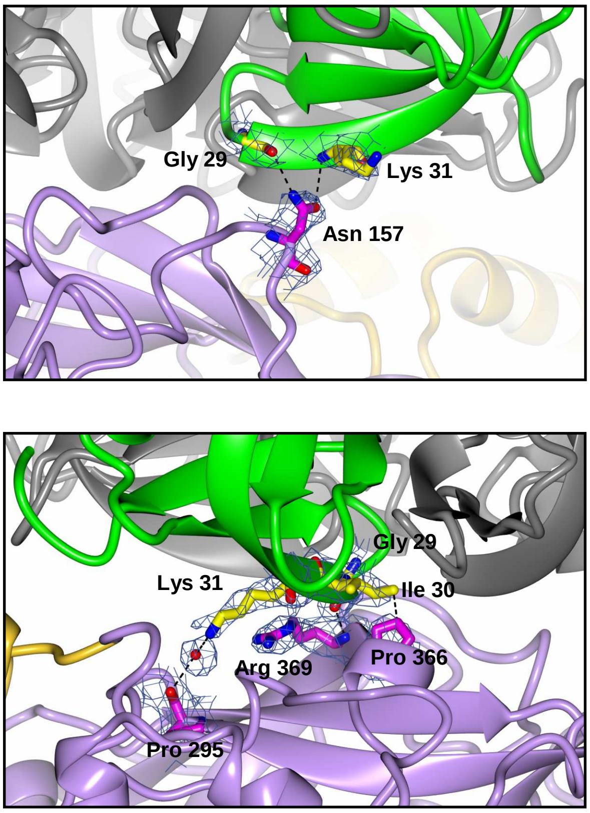

Fig. S4. Interactions of Hfq subunits 2 and 6 with HPII. (A) Subunit 2 in green. (B) Subunit 6 in green. The figures display the interface between the distal surface of Hfq and the core domain of HPII. The representation scheme of molecules is the same as in Fig. 5. See also Table S2 for more information on the interactions.

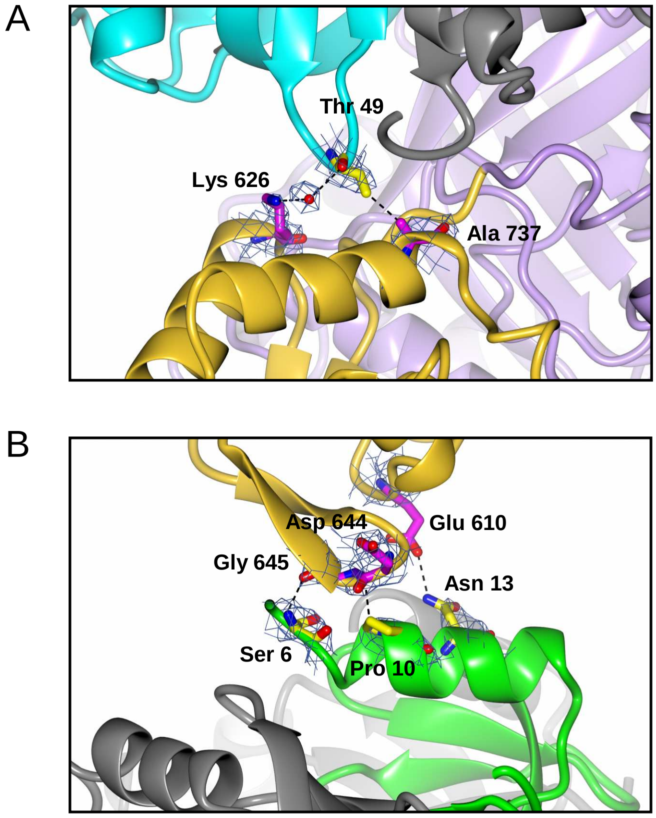

Fig. S5. Interactions of Hfq subunits 1 and 4 with HPII. (A) Interface between the distal surface of Hfq subunit 1 (cyan) and the C-terminal lobe of HPII. (B) Interface between the proximal surface of Hfq subunit 4 (green) and lower part of the C-terminal lobe of HPII in a neighboring tetramer. The representation scheme is the same as in Fig. 5. Densities for the side chains of Glu 610 are not visible at this contour level.
